# Defining Human Embryo Phenotypes by Cohort-Specific Prognostic Factors

**DOI:** 10.1371/journal.pone.0002562

**Published:** 2008-07-02

**Authors:** Sunny H. Jun, Bokyung Choi, Lora Shahine, Lynn M. Westphal, Barry Behr, Renee A. Reijo Pera, Wing H. Wong, Mylene W. M. Yao

**Affiliations:** 1 Department of Obstetrics and Gynecology, Stanford University School of Medicine, Stanford, California, United States of America; 2 Department of Applied Physics, School of Humanities and Sciences, Stanford University, Stanford, California, United States of America; 3 Center for Human Embryo and Embryonic Stem Cell Research and Education, Institute for Stem Cell Biology & Regenerative Medicine, Stanford University, California, United States of America; 4 Department of Statistics, School of Humanities and Sciences, Stanford University, Stanford, California, United States of America; Vanderbilt University Medical Center, United States of America

## Abstract

**Background:**

Hundreds of thousands of human embryos are cultured yearly at *in vitro* fertilization (IVF) centers worldwide, yet the vast majority fail to develop in culture or following transfer to the uterus. However, human embryo phenotypes have not been formally defined, and current criteria for embryo transfer largely focus on characteristics of individual embryos. We hypothesized that embryo cohort-specific variables describing sibling embryos as a group may predict developmental competence as measured by IVF cycle outcomes and serve to define human embryo phenotypes.

**Methodology/Principal Findings:**

We retrieved data for all 1117 IVF cycles performed in 2005 at Stanford University Medical Center, and further analyzed clinical data from the 665 fresh IVF, non-donor cycles and their associated 4144 embryos. Thirty variables representing patient characteristics, clinical diagnoses, treatment protocol, and embryo parameters were analyzed in an unbiased manner by regression tree models, based on dichotomous pregnancy outcomes defined by positive serum ß-human chorionic gonadotropin (ß-hCG). IVF cycle outcomes were most accurately predicted at ∼70% by four non-redundant, embryo cohort-specific variables that, remarkably, were more informative than any measures of individual, transferred embryos: Total number of embryos, number of 8-cell embryos, rate (percentage) of cleavage arrest in the cohort and day 3 follicle stimulating hormone (FSH) level. While three of these variables captured the effects of other significant variables, only the rate of cleavage arrest was independent of any known variables.

**Conclusions/Significance:**

Our findings support defining human embryo phenotypes by non-redundant, prognostic variables that are specific to sibling embryos in a cohort.

## Introduction

Developmental arrest of human embryos cultured *in vitro* is common and presents a major obstacle to achieving pregnancy through IVF, as well as a major obstacle to research in human embryonic stem cell (hESC) biology [1], [2], [3], [4], [5], [6], [7]. While the culture of embryos to blastocyst stage for subsequent transfer yields high pregnancy rates and minimizes the risk of multiple gestation, the availability of blastocysts is limited even in the best IVF clinics because of the high rates of attrition in *in vitro* embryo culture [1], [3], [4], [8], [9], [10], [11].

Although developmental defects such as cleavage arrest, polyploidy, and fragmentation are commonly encountered and have been used for scoring individual embryos in IVF [11], [12], [13], [14], [15], [16], [17], [18], [19], [20], [21], the lack of well-defined human embryo phenotypes has hindered translational research and mechanistic investigations. One key challenge to defining human embryo phenotypes relates to the unclear and often highly interactive relationships amongst variables pertaining to patient characteristics, clinical infertility diagnoses, IVF treatment protocols, and observed embryo characteristics. Further, since any single couple may typically produce a few oocytes or embryos that are abnormal merely by chance, it is difficult to determine whether sibling embryos as a group, or an embryo cohort, is “normal”. (Note that “embryo cohort” refers to an embryo sibling group from the same couple within the same IVF treatment.) Nonetheless, we envision that the identification of cohort-specific parameters to define human embryo phenotypes is a necessary step towards translational investigations of molecular determinants of developmental competence. Thus, we sought to test the hypothesis that embryo cohort-specific variables have prognostic value in measuring IVF cycle outcomes by identifying non-redundant, prognostic variables in an unbiased manner using regression tree models.

## Results

**Figure 1:**
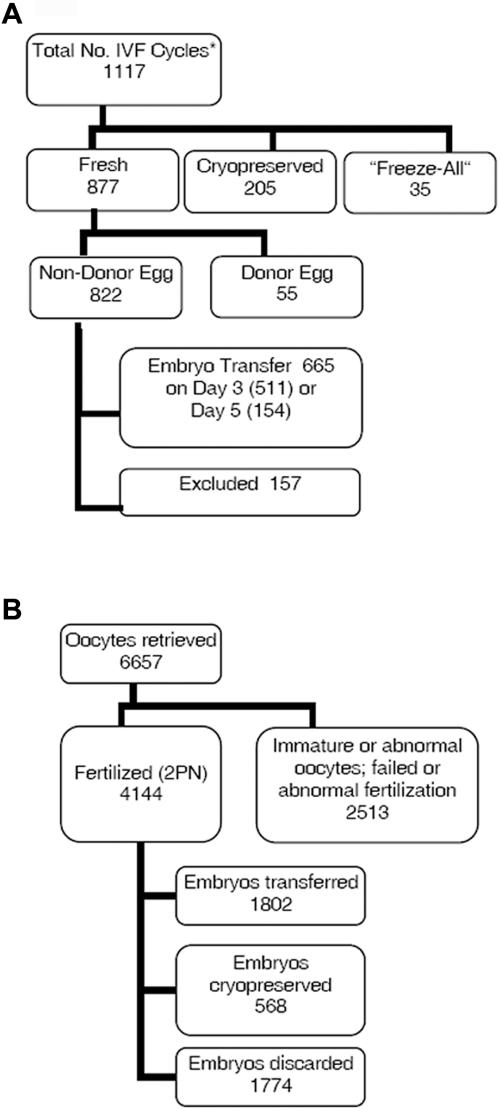
Source of data. A) IVF cycles performed in 2005. B) Utilization of oocytes and embryos in 665 fresh, non-donor IVF cycles. * All numbers in Panel A indicate the number of cycles and numbers in Panel B indicate the number of oocytes or embryos. Fresh cycles are defined by ovarian stimulation of gonadotropins and embryo transfer performed within the same cycle; cryopreserved cycles utilize embryos that were obtained and cryopreserved from a previous cycle; “freeze-all” are cycles in which ovarian stimulation was performed, but embryos were cryopreserved instead of being transferred back within the same cycle for medical or non-medical reasons. 157 cycles were removed from analysis for a variety of medical and non-medical reasons that did not result in fresh embryo transfer (see *SI Text* for details).

**Figure 2:**
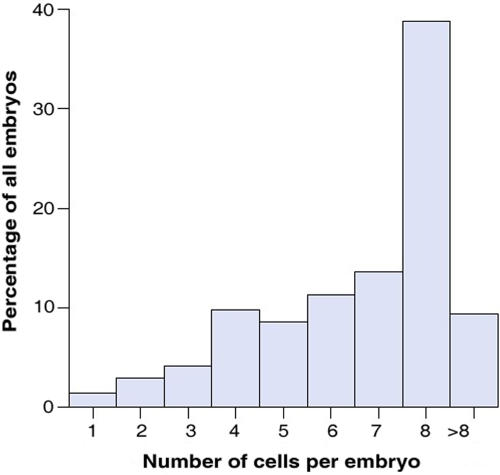
Distribution of all embryos from 665 fresh, non-donor IVF cases according to their cell number on Day 3.

Of all 1117 IVF treatments performed at Stanford University in 2005, 822 were fresh IVF cycles that used the patients' own oocytes (Figure 1A). Based on our exclusion criteria, 157 cycles were excluded for a variety of medical and non-medical reasons (see results in *Supporting Information Text S1* for details). Clinical and embryology data on the remaining 665 cycles that satisfied inclusion and exclusion criteria, and their 4144 embryos, respectively, were analyzed to test the hypothesis that cohort-specific variables predict IVF cycle outcomes (Figure 1). Of those 4144 embryos, the number of blastomeres or cells on day 3 was recorded for 4002 embryos (96.6%). Overall, 38.8% had 8 cells, the developmentally appropriate cell number, while 18.2% of embryos had ≤4 cells, and 33.6% had 5–7 cells (Figure 2).

### Prognostic Significance and Correlation of Variables

We systematically examined the association of each variable with IVF outcomes, and the correlation of each pair of variables. Pair-wise logistic regression tests confirmed many known prognostic variables, including female age, day 3 FSH, and the number of 8-cell embryos. However, in addition to these known prognostic variables, we observed that cohort-specific variables such as fertilization rate and the rate of cleavage arrest were also significantly associated with IVF cycle outcome (p<0.001; Table 1). In contrast, except for male factor infertility (p<0.05), none of the conventional clinical infertility diagnoses were significantly associated with IVF outcomes. Notably, despite a high degree of correlation between many variables and age or day 3 FSH level, which estimates ovarian aging, neither age nor day 3 FSH level was correlated to cohort-specific embryo parameters (see Table S1). Collectively, these results suggest that determinants other than age-related mechanisms and clinical diagnoses impact cohort-specific embryo developmental competence.

**Table 1:** Association of each variable with pregnancy outcome.

Variables	Estimate*	S.E.	P-Value
**Patient Characteristics and Clinical Diagnoses** †			
Age	−0.10	0.02	2.16E-007
Maximum Day 3 FSH level	−0.08	0.03	1.70E-003
Gravidity	0.036	0.066	5.86E-001
Male Factor (infertility diagnosis)	0.50	0.24	3.71E-002
**IVF Cycle Characteristics**			
Microdose lupron (flare) protocol	−1.14	0.24	2.53E-006
Antagonist protocol	−0.74	0.19	9.98E-005
Performance of ICSI	−0.15	0.16	3.47E-001
No. of oocytes	0.08	0.01	1.58E-009
**Embryo Cohort Parameters**			
Fertilization rate	1.24	0.36	5.37E-004
No. of embryos	0.14	0.02	2.67E-012
Average cell no. of embryos	0.29	0.06	6.34E-006
No. of 8-cell embryos	0.26	0.04	2.88E-012
Percentage of 8-cell embryos	0.76	0.28	5.75E-003
Cleavage arrest rate‡	−1.28	0.35	2.76E-004
Average grade of embryos	−0.091	0.17	5.88E-001
**Parameters of Transferred Embryos**			
Day 5 embryo transfer§	1.40	0.19	7.51E-013
No. of embryos transferred	0.0058	0.053	9.12E-001
Average cell no. of embryos transferred	0.47	0.07	2.19E-010
Percentage of transferred embryos at the 8-cell stage	1.33	0.21	5.35E-010
No. of 8-cell embryos transferred	0.41	0.08	4.40E-008
No. of embryos with ≤4 cells transferred	−2.14	0.49	1.06E-005
Average grade of embryos transferred	−0.52	0.17	2.61E-003

* Positive and negative estimates indicate association with positive and negative pregnancy outcomes, respectively.

† Clinical infertility diagnoses that were not significantly associated with pregnancy outcome (p-value >0.05) were not listed: uterine factor, polycystic ovarian syndrome, endometriosis, tubal ligation, tubal disease, hydrosalpinges, unexplained infertility, and “other diagnoses”. Each IVF case may have more than one clinical infertility diagnosis.

‡ Cleavage arrest rate is defined as the percentage of embryos with 4 or fewer cells on Day 3 of *in vitro* culture.

§ Day 5 embryo transfer is arbitrarily listed under Parameters of Transferred Embryos. It can also be considered an Embryo Cohort Parameter, as it depends on the total number of embryos and the number of 8-cell embryos.

### Thresholds of Non-redundant, Prognostic Variables Defining Human Embryo Cohort Phenotypes

Sequential Multiple Additive Regression Tree (MART®) and Classification and Regression Tree (CART) analyses of all 30 variables (listed in Table 1 and its legend) determined that IVF cycle outcomes were most accurately predicted at ∼70% by using only four non-redundant variables: total number of embryos, rate of cleavage arrest in an embryo cohort, the number of 8-cell embryos in a cohort, and day 3 FSH level. Remarkably, these four variables all describe the embryo cohort rather than individual embryos, and were more informative than age, clinical diagnoses, or any measures of the transferred embryos. Interestingly, the total number of embryos, day 3 FSH, and the number of 8-cell embryos depended on and thus captured the effects of many other variables. In contrast, the rate of cleavage arrest was independent of any of those known variables. (Details on MART® and CART analyses are reported in Text S1, and Figure S1)

Of the prognostic thresholds identified, the most robust phenotypes are A1 and A2, and B1 and B2 (Table 2). Number of embryos <6 or ≥6 is used by all 5 top CART models, defines all other phenotypes (B to F), and can be applied to all cases. Specifically, the phenotype defined by having fewer than 6 embryos, has an odds ratio of 3.9 for no pregnancy compared to cases with ≥6 embryos (95% Confidence Interval [CI], 2.8 to 5.5). Similarly, the next most robust phenotypes are defined by the number of embryos and cleavage arrest rate, such that for cases with ≥6 embryos, those with cleavage arrest rate >14.6% are 3.0 times more likely to result in no pregnancy than those with cleavage arrest rate ≤14.6% (95% CI, 1.9 to 4.9).

**Table 2:** Prognostic thresholds defining cohort-specific phenotypes.

	Embryos (No.)*	Cleavage Arrest (%)*	8-cell embryo (No.)*	FSH (mIU/mL)*	Pregnancy-No. (%)†	No Pregnancy-No. (%) ‡	Applicable Cases–No. (%) §	No. Trees ¶	Reference Condition∥	Odds Ratio	95% Confidence Interval (C.I.)
A1**	≥6				177 (57.7)	130 (42.3)	307 (46.2)	5			
A2	<6				92 (25.7)	266 (74.3)	358 (53.8)	5	A1	3.9	(2.8, 5.5)
B1	≥6	≤14.6			112 (70.4)	47 (29.6)	159 (23.9)	4			
B2	≥6	>14.6			65 (43.9)	83 (56.1)	148 (22.3)	4	B1	3.0	(1.9, 4.9)
B3	≥6	14.6–52.8			62 (47.3)	69 (52.7)	131 (19.7)	1	B1	2.6	(1.6, 4.3)
B4	≥6	≤52.8			174 (60.0)	116 (40.0)	290 (43.6)	n/a			
B5	≥6	>52.8			3 (17.6)	14 (82.3)	17 (2.6)	1	B1	10.6	(3.2, 49.6)
									B4	6.7	(2.1, 30.9)
C1	≥6		≥2		157 (63.6)	90 (36.4)	247 (37.1)	1			
C2	≥6		<2		20 (33.3)	40 (66.7)	60 (9.2)	1	C1	3.5	(1.9, 6.4)
D1	≥6	>14.6	≥2		51 (53.1)	45 (46.9)	96 (14.4)	1			
D2	≥6	>14.6	<2		14 (26.9)	38 (73.1)	52 (7.8)	1	D1	3.0	(1.5, 6.5)
E1	≥6	>14.6	≥2	≤4.6	14 (82.4)	3 (17.6)	17 (2.6)	1			
E2	≥6	>14.6	≥2	>4.6	34 (46.9)	37 (53.1)	71 (12.2)	1	E1	4.8	(1.4, 23.4)

* Cohort phenotypes defined by thresholds of non-redundant prognostic variables. Each set of conditions (A–E) use “AND” as the operator where more than one condition is listed.

† No. of cases that satisfy the threshold conditions and have pregnancy outcome. This percentage is calculated by using the No. Applicable Cases as denominator. In general, conditions that discriminate between pregnancy and no pregnancy outcomes more highly are more robust and are expected to be more useful in both clinical management and translational research.

‡ No. of cases that satisfy the threshold conditions and have no pregnancy outcome. This percentage is calculated by using the No. Applicable Cases as denominator.

§ The No. Applicable Cases is the total number of cases that satisfy the threshold conditions. This percentage is calculated by using the total number of cycles (665) as the denominator. In general, the larger the number of applicable cases, the more useful the set of conditions are for clinical management and counseling. However, for the purpose of translational research, conditions that define a smaller number of cases may have more specific correlates on a molecular level.

¶ No. Trees shows the number of CART trees that utilize each set of conditions. There are a total of 5 trees. (See Supplemental Results.) Increased utilization indicates “usefulness” or “robustness” of that particular set of conditions.

∥ Reference condition against which the Odds Ratio and 95% C.I. for having no pregnancy is calculated.

** Conditions A–E are listed from most robust and “useful” to least “useful” based on: the number of trees that utilize each set of conditions, the number of applicable cases, and the odds ratio and 95% CI.

In contrast, the rest of the thresholds listed in Table 2 are used by only 1 CART model each, and is applicable to fewer cases. However, as some of those phenotypes describe very specific subset of cases and have odds ratios that are highly discriminatory, they may be extremely useful depending on the clinical or translational research context. For example, for cases with ≥6 embryos, having cleavage arrest rates of 14.6–52.8% and >52.8% increase the odds of no pregnancy by 2.6 (95% CI 1.6 to 4.3) and 10.6 (95% CI 3.2 to 49.6), respectively, when compared to cases with cleavage rates of ≤14.6%.

## Discussion

Since the introduction of IVF in the 1970s, the major challenges of assisted reproductive technologies (ART) have been the high attrition rates of embryos cultured *in vitro* [1], [3], [4], [8], [9], [10], [11], [22], [23], the limited value of embryo morphology in predicting developmental competence [24], [25], [26], and finding criteria to help determine the number of embryos to transfer [22], [23]. In addition, the benefit of aneuploidy screening by preimplantation genetic screening (PGS) has recently been refuted [27]. Thus, there is a need to reassess factors that determine human embryo quality.

Our findings represent a first step towards this goal by using regression tree models, MART® and CART, as unbiased methods to analyze IVF and embryo data. These methods allowed us to consider and control for a large number of variables, even if only a few of them have significant impact on outcomes. This feature is critical for the analysis of the highly interactive and multicollinear IVF and human embryo data, as arbitrary selection of variables may compromise completeness of data and introduce bias, while including all of them would cause the conventional multivariate regression to breakdown (see *SI Text*). Indeed, such application of CART analysis was taken by Guzick *et al.* to define semen parameters that predicted male infertility [28]. In our study, we further used MART®, a more powerful statistical method that “boosts” or increases accuracy in the CART method [29], [30], [31], [32].

We identified four non-redundant variables that predict outcomes in the current IVF cycle with ∼70% accuracy. Most remarkably, these variables–total number of embryos, cleavage arrest rate, number of 8-cell embryos, and day 3 FSH (in order of relative importance)–describe the entire embryo cohort, and are more predictive than any measures of the transferred embryos. In addition, we show that most prognostic information carried by highly interacting and multicollinear conventional variables such as age and clinical diagnoses, is captured by three of the four variables.

Previous reports mainly focused on the prognostic value of individual embryo scores, in which the relative weighting of score components was determined arbitrarily rather than by objective or statistical methods [15], [18]. Further, although individual variables that were significantly related to IVF cycle outcomes were reported, there has been no attempt to compare their relative prognostic value, or to identify redundancy amongst variables [12], [14], [17], [21]. For example, age, serum FSH, number of oocytes and number of embryos were each reported to be significantly related to IVF outcomes [17]. However, as shown by our analyses, the prognostic value of age and number of oocytes was captured by three of the four non-redundant variables. Similarly, the total number of embryos and the number of 8-cell embryos have been advocated for use in selecting patients for blastocyst transfer in some IVF clinics to minimize the risk of having no embryos to transfer due to failed blastocyst development [21], [33], [34]. However, the prognostic value of these two variables has not been compared to that of others, and their ability to capture prognostic information from most other variables were not known.

Indeed, cleavage arrest rate is the only variable that is independent of the others, which suggests that it may be linked to biological mechanisms that are not currently recognized in the management of clinical infertility or hESC biology. Encountered in ∼18% of human embryos cultured *in vitro* overall, its underlying defects are likely diverse, and may be due to suboptimal *in vitro* culture environment, biological mechanisms underlying infertility, a generally poor reproductive fitness of our species or all of these factors. Although cleavage arrest coincides with the maternal-embryonic transition during which maternal transcripts are degraded and the embryonic genome is activated [35], gene expression analyses of arrested single human embryos did not show failure in embryonic genome activation, and no specific molecular defects have been identified [8], [11].

Our study has some limitations. Although we took advantage of the power of regression tree models to analyze a very comprehensive range of variables, we did not include cryopreservation of sibling embryos and assisted hatching as variables. In addition, it would also be valuable to analyze blastocyst development rate of sibling embryos, because this variable has been shown to correlate with positive pregnancy outcomes [36]. Those variables are now being investigated in a larger study that encompasses four years of data. As the goal of this current study was to explore new paradigms in human embryo development in IVF, and not to arrive at recommendations to change clinical practice, we used positive serum hCG status as the surrogate outcome measure to identify nonredundant predictors of IVF cycles in which at least one embryo attaches to the endometrium and secretes hCG, from those in which no embryo attaches. In the future, we will use later endpoints, such as clinical pregnancy or live birth, to address clinical questions.

In spite of over 30 years of ART, many challenges remain. Ongoing and future investigations may incorporate approaches common to genetics and developmental biology, in order to reassess defective human embryo development in terms of phenotypes that can be diagnosed, defined, and translated into improved clinical practices. Collectively, our results indicate that embryos from a cohort share as yet undefined genetic or epigenetic determinants of developmental competence, which is consistent with the greater increase in implantation relative to pregnancy rates conferred by blastocyst transfer [37]. The concept of cohort-specific determinants suggest a paradigm shift from strictly focusing research efforts on selecting the “best” embryos to identifying methods that would improve the quality of the entire cohort. In addition, it raises the question of whether quality of the entire cohort is intrinsic due to the shared origins of the embryos, or if it is merely a result of group culture *in vitro*, especially since the benefits of group culture have been reported in animal and human embryos [38], [39], [40], [41]. While embryo-specific parameters may help to identify embryos that would maximize the immediate pregnancy outcome for each couple, in the long term, understanding cohort-specific parameters is critical in counseling patients, improving treatment, and ultimately in developing mechanism-specific and more customized treatments.

We reason that well-defined criteria for embryo cohort phenotypes in selecting abnormal embryos for molecular analyses would maximize the chance of finding non-random genetic or epigenetic molecular defects that are consistent in an embryo cohort. For example, we are applying our findings to analyze arrested embryos from embryo cohorts in which the number of embryos are ≥6 and cleavage arrest rate is >52.8% (see Condition B5 in Table 2). Overall, ∼2.5% of fresh, non-donor IVF cases (or ∼17 cases per year, at our center) are expected to fulfill these criteria. This approach should allow for objective interpretation and comparison of data both internally and amongst research groups.

We are also applying this research strategy to investigate predictors of pregnancy outcomes in subsequent IVF cycles to contrast couple- versus embryo cohort-specific prognostics variables. More importantly, new hypotheses that are generated by this investigation can be further tested as additional years of data become available. For example, our findings indicate that a low day 3 FSH (<4.6 mIU/mL) confer high pregnancy rates in a very small and specific subset of patients (see Condition E in Table 2), and offer new perspectives on this controversial entity. While abnormally high levels of day 3 FSH have been associated with ovarian aging, poor ovarian response in IVF, and poor IVF cycle outcomes, many studies have cautioned against its use in clinical management due to its low sensitivity, especially in women under 40 [42], [43], [44], [45]. However, the clinical utility of this test may be improved by determining appropriate thresholds and conditions [46].

Similar to the implications for ART, our results also raise questions about the effects of cohort-specific determinants on the success rate of hESC line derivation, the quality of hESC lines, and most importantly, embryo cohort selection for hESC line derivation, or oocyte cohort selection for somatic cell nuclear transfer. Currently, most scientific reports on successful derivation of hESC lines do not include information on embryo cohort characteristics, clinical information or IVF outcomes of sibling embryos. Our findings suggest that correlation of clinical IVF data and hESC line characteristics may provide valuable insight that would move both the fields of reproductive medicine and hESC research forward. We envision that dissection of human embryo phenotypes and their corresponding molecular correlates is not only a necessary step towards improving the treatment of clinical infertility, but will also contribute significantly to research efforts in the hESC field.

## Materials and Methods

### Data Collection, Inclusion and Exclusion Criteria

Data related to clinical diagnoses, IVF treatment protocol and monitoring, embryology data and treatment outcomes for all IVF cycles performed between January 1, 2005 and December 31, 2005 at Stanford University Medical Center were retrieved from BabySentryPro (BabySentry Ltd, Limassol, Cyprus), a widely used fertility database management system, or obtained from medical and embryology records as necessary. Retrospective data collection, de-identification, and analysis were performed according to a Stanford University Institutional Review Board-approved protocol. The inclusion criteria for data analysis were fresh, stimulated, non-donor oocyte IVF cycles. We excluded cycles that did not result in embryo transfer for any reason, cycles performed for women aged over 45, and those performed for preimplantation genetic screening.

### Assessment of Embryo Development

Our standard clinical protocols for ART treatment, fertilization, embryo culture, embryo assessment, cryopreservation criteria, and clinical outcomes are described in methods in *SI Text*. The normal progression of human embryo development *in vitro* is characterized by the appearance of 2 pronuclei at 16–20 hours after insemination as evidence of fertilization on Day 1, with Day 0 as the day of oocyte retrieval. By late Day 1, embryo development has reached the 2-cell stage, followed by the 4-cell and 8-cell stages on Days 2 and 3, respectively. On Days 4 and 5, embryo development is characterized by the establishment of the morula and blastocyst stages, respectively. All embryos were available for evaluation on Day 3. The day of embryo transfer was determined by the number of blastomeres on Day 3. In general, if 4 or more 8-cell embryos were present, we would recommend extended embryo culture until Day 5, when blastocyst transfer, which has been associated with higher pregnancy rates, would be performed. If fewer than four 8-cell embryos were present, embryo transfer would be performed on Day 3.

### Patient, IVF Cycle, and Embryo Parameters

We analyzed 30 variables for association with IVF treatment outcomes, as listed in Table 1, under four main categories: patient characteristics and clinical diagnoses, IVF cycle characteristics, embryo cohort parameters, and parameters of transferred embryos. The cleavage arrest rate was defined as the percentage of embryos within a cohort with 4 or fewer cells on Day 3 of *in vitro* culture. All other variables were self-explanatory.

### Statistical Analysis

Since some patients underwent more than one IVF cycle during the study period, the analyses were performed based on treatment cycles rather than patients. Statistical analyses were performed based on the dichotomous outcomes of no pregnancy, as defined by negative serum ß-hCG, and pregnancy, as defined by positive serum ß-hCG, and included biochemical pregnancy, clinical pregnancy, spontaneous abortion, and ectopic pregnancy. We performed pair-wise logistic regression of each variable to the outcome and determined the Pearson correlation coefficient between each pair of continuous variables.

For the main analyses, boosted classification trees were constructed by MART® to identify non-redundant prognostic variables, which were then further analyzed by CART to identify thresholds that would define them as categorical variables. MART® is a robust method used to identify interactive structure of variables that are predictive of outcomes [29], [30], [31], [32]. The use of cross-validation and boosting in parameter selection and model assessment in MART® also preserve parsimony and prevent over-fitting [31]. In the MART® tree constructions, the whole data set is divided into 10 subsets to achieve 10 fold cross validation for model assessment. The same 10 fold cross validation was repeated 1000 times to perform a robust prediction rate estimation and identify tree models with the highest prediction rates in the CART. While MART® is powerful in selecting non-redundant prognostic variables from a large set of highly interactive variables, CART analysis results in simple algorithms, and more easily understood “decision trees”, that are used in the medical literature [28]. Thus non-redundant, prognostic variables identified by MART® to confer prediction were analyzed by CART to further define prognostic thresholds.

## Supporting Information

Text S1 This SI file contains details pertaining to methods, results, and statistical analyses which may be of interest to certain readers. It also contains an SI Table 2.
(0.10 MB DOC)

Table S1 Correlation between each pair of variables.
(0.08 MB DOC)

Figure S1 Variables and their relative importance in determining A) number of 8-cell embryos, B) day 3 FSH, and C) the total number of embryos.
(2.65 MB TIF)
