# Supplementary material for: Defining Human Embryo Phenotypes by Cohort-Specific Prognostic Factors
Source: PLoS One. 2008 Jul 2;3(7):e2562. doi: 10.1371/journal.pone.0002562 (PMC2432022; doi:10.1371/journal.pone.0002562)
Supplement: Text S1 — This SI file contains details pertaining to methods, results, and statistical analyses which may be of interest to certain readers. It also contains an SI Table 2. (0.10 MB DOC) [file pone.0002562.s001.doc]

**Supporting Information (SI)**

**METHODS**

**Treatment Protocols used for ART**

The majority of our analyzed IVF cycles were performed in patients with poor ovarian reserve, severe male factor infertility, tubal infertility, anovulatory disorders, or unexplained etiology. In general, one of three stimulation protocols was used in each treatment cycle: luteal downregulation (long) was used for most patients, and microdose lupron (flare) and antagonist protocols were used primarily for patients with presumed diminished ovarian reserve or with a history of previously failed IVF cycles. The long protocol consisted of luteal downregulation using 0.5 mg leuprolide acetate, which was decreased to 0.25 mg with stimulation. In the flare protocol, microdose lupron (0.04 mg s.c. bid) was started after 2-4 weeks of oral contraceptive pills. In the antagonist protocol, GnRH antagonist was initiated when the lead follicle reached 14mm in size. In all three protocols, baseline ultrasound testing was performed to document that no cysts > 1.5 cm were present in the ovaries. When baseline criteria were met, gonadotropin therapy using recombinant FSH with human menopausal gonadotropin was begun. Stimulation was generally achieved using daily dosing of a total of 150-600 IU per day in order to maximize follicular recruitment. Ultrasound monitoring of follicular growth was performed starting on cycle day 7 and then every 1-3 days as indicated. Serum estradiol levels were monitored as necessary.

A dose of 10,000 IU of human chorionic gonadotropin was administered when at least two follicles reached an average diameter of >17mm. Transvaginal ultrasound-guided oocyte retrieval was performed 34-36 hours after hCG administration in the standard fashion with monitored anesthetic care.

**Oocyte Fertilization and Embryo Culture**

Oocytes were cultured in groups before fertilization under mineral oil in approximately 125 μl droplets of Sage Cleavage Medium (Cooper Surgical, Inc, Trumbull, CT) with 10% Serum Protein Substitute (SPS, Irvine Scientific, Santa Ana, CA). Oocytes destined for conventional IVF were cultured in groups of 5 and oocytes destined for intracytoplasmic sperm injection (ICSI) were cultured in groups of up to 20 after stripping the cumulus cells. Oocytes were inseminated with sperm if the semen analysis was normal and fertilization was expected to be normal. Oocytes were inseminated conventionally between 4-6 hours after retrieval. If the semen analysis was abnormal or poor fertilization was expected, then the oocytes were injected with sperm using ICSI. The fertilized oocytes were cultured in groups of up to 20 under mineral oil in approximately 125 μl droplets of Sage Cleavage Medium (Cooper Surgical, Inc, Trumbull, CT) with 10% SPS at 37°C in a humidified atmosphere of 5% O_2_, 5% CO_2_ and 90% N_2_. Fertilization check was performed 16~18 hours after insemination or ICSI. The zygotes with clear two pronuclei were cultured for another 48 hours in Sage Cleavage Medium with 10% SPS. Oocytes with single pronucleus (1PN) or three or more pronuclei were considered abnormally fertilized. If Day 5 blastocyst transfer was indicated, extended embryo culture was be performed in Quinn’s Advantage Blastocyst medium (Cooper Surgical) with 10% SPS for 48 hours before transfer. Of note, the same culture media was used during the study time period.

**Cleavage and Grading of Embryos**

A single team of experienced embryologists evaluated the embryos on post-retrieval day 3, 68 to 72 hours after oocyte harvest. Embryos were examined for cleavage (cell number) and grade, which includes cytoplastmic fragmentation. Embryos were graded as follows on Day 3: Grade 1, blastomeres have equal size and no cytoplasmic fragmentation; Grade 2, blastomeres have equal size and minor cytoplasmic fragmentation involving < 10% of the embryo; Grade 3, blastomeres have unequal size and fragmentation involving 10-20% of the embryo; Grade 4, blastomeres have equal or unequal size, and moderate to significant cytoplasmic fragmentation covering 20-50% of the embryo; and Grade 5, few blastomeres and severe fragmentation covering ≥50% of the embryo [1]. Of note, cytoplasmic fragmentation in embryos was easily differentiated from cleavage based on the size of the fragments, their location within the embryo, and the absence of a nucleus. In contrast, cytoplasmic fragmentation of oocytes was not included as a variable because it was extremely rare. The presence or absence of compaction was routinely noted by our embryologists on Day3. As compaction was observed in <10% of embryos, we did not include this variable in the analysis.

**Assisted Hatching**

Indications for assisted hatching (AH) in our center were advanced maternal age, elevated FSH level, and/or a history of multiple failed assisted reproduction cycles. On the day of embryo transfer, embryos for hatching were placed in phosphate-buffered saline (PBS) with 10% SPS. AH was accomplished by using the ZILOS-tk laser (Hamilton Thorne Biosciences, Beverly, MA) to make a hole in an area of the zona pellucida that was between blastomeres. Embryos were then rinsed and returned to the media until transfer.

**Embryo Transfer and Cryopreservation**

Ultrasound-guided embryo transfer was performed using a Tefcat or Echotip Softpass catheter (Cook Ob/Gyn, Spencer, IN). Progesterone supplementation with vaginal suppositories was performed in all patients. For patients having embryo transfer on Day 3, any remaining embryos with more than five blastomeres were placed in extended culture for another 2 or 3 days. Any expanding, expanded and hatching blastocysts with good inner cell mass and trophectoderm were frozen on day 5 or day 6. In addition, cryopreservation was performed on excess embryos, severe ovarian hyperstimulation syndrome, and fertility preservation due to medical or social reasons. Excess embryos that were not transferred were commonly discarded if they were not of sufficient quality for cryopreservation or if patients did not opt for embryo cryopreservation due to non-medical reasons. In cases of pre-implantation genetic diagnosis, embryos that tested positive for genetic diseases or aneuploidy were also discarded.

**Clinical Outcomes**

Serum quantitative ß-hCG levels were obtained at 8-10 days after embryo transfer, and followed serially until the diagnosis of clinical pregnancy was made by the presence of a gestational sac on transvaginal ultrasound. Outcomes other than clinical pregnancy included: 1) no pregnancy if serum quantitative ß-hCG was negative; 2) biochemical pregnancy as defined by decreasing serum quantitative ß-hCG levels before a gestational sac could be visualized by transvaginal ultrasound; 3) spontaneous abortion as defined by pregnancy loss after a gestational sac was visualized by transvaiginal ultrasound; and 4) ectopic pregnancy; and 5) other abnormal gestations such as gestational trophoblastic disease. Live birth outcomes were obtained by follow-up contact with patients as part of routine clinical care, but they were not used in this study.

**REGRESSION TREES**

Clinical IVF data, especially when considering oocyte and embryo parameters, often do not lend themselves to meaningful analysis by multivariate logistic regression. The high degree of interaction and multicollinearity of many relevant variables interfere with conventional multivariate regression. In these situations, the regression and classification tree models (CART), has been widely used in clinical research [2,3,4,5,6,7,8]. Here, we used Multiple Additive Regression Tree (MART®), a more powerful statistical method that combines “boosting” with CART to “boost” or increase accuracy in the CART method, to identify non-redundant prognostic variables.

In general, regression trees have several key advantages: 1) ability to consider all types of clinical IVF and embryology data, including numeric, ordinal, binary, and categorical variables; 2) ability to handle missing values well based on a “surrogate” splitting technique without the need for imputation; 3) ability to generate results that are invariant to monotonic data transformation and thus eliminate the need to test different methods of data transformation or metrics; 4) ability to generate trees that are immune to the effects of extreme outliers; 5) ability to generate trees that inherently explore and identify interactions of variables that would otherwise need to be explicitly stated in a multiple logistic regression. Most importantly, regression trees can consider a large number of variables, including ones that may turn out to be irrelevant, even if only a small number of variables have significant statistical impact on outcomes. This ability to consider many variables is critical for analysis of IVF outcomes, as many variables, such as percentage of 8-cell embryos, number of 8-cell embryos, percentage of 8-cell embryos transferred, and number of 8-cell embryos transferred, may be highly interactive; thus, arbitrarily selecting one of them may compromise completeness of data and introduce bias, while including all of them may cause the conventional multivariate regression to breakdown.

The results from MART® may help to identify variables that could be re-analyzed by multivariate logistic regression. Often, they may identify thresholds, or “cut-offs”, that will be used to create categorical variables to segregate cases into subgroups for further inter-group comparison of characteristics by conventional methods such as t-tests, chi-square analysis, or Wilcoxon rank sum test. For example, CART analysis was used by Guzick et al. to classify men as subfertile, of indeterminate fertile status, or fertile based on threshold values for sperm concentration, motility, and morphology, exemplifies the power of this strategy in infertility research [7].

**RESULTS**

**REASONS FOR THE EXCLUSION OF 157 IVF CASES**

The 157 cycles that were excluded consisted of: cancelled oocyte retrieval due to poor ovarian stimulation (63 cycles), cancelled embryo transfer due to complete lack of embryo development (8 cycles), cancelled embryo transfer due to unexpected medical or non-medical reasons (35 cycles), cycles that were not treated with gonadotropins (3 cycles), missing outcomes (8 cycles), and women being ≥ 45 years of age based on age alone (29 cycles). In our study, 160 patients underwent subsequent repeat cycles after a previously failed attempt for a total of 368 cycles. Of these, 126 patients had 2 IVF cycles performed, 25 patients had 3 cycles, 6 patients had 4 cycles, 1 patient had 5 cycles and 2 patients had 6 cycles performed in the same year at the same institution. 511 cycles (76.8%) with Day 3 embryo transfer and 154 cycles (23.2%) with Day 5 transfer, or a total of 665 IVF cycles, fulfilled the inclusion and exclusion criteria for analyses.

**ANALYSES BY MART® AND CART MODELS**

To overcome the challenges presented by the highly interactive nature and potentially non-linear dependence amongst variables, and their multicollinearity, all 30 variables listed in Table 3 and its legend were analyzed by MART® to generate models that utilize non-redundant prognostic variables. Eight models were generated by MART® with increasing complexity of individual trees for boosting, while the learning rate was chosen to produce a minimum test error for the given complexity. Among them, the 3 top-ranking models had cross-validated (CV) prediction error rates of 0.301 to 0.308, which separated them well from the other five models, whose CV error rates ranged from 0.315 to 0.331 (SI Table 2). The third model, however, was based on very complex individual trees yet showed a larger CV error than the two preceding models; thus it was excluded from the analysis. The resulting two top models, consistent with characteristics of meaningful regression tree models, used very few trees and shared common features, such that the two models collectively used only 5 trees containing 5 variables, while some of the other models used up to 16 to 30 trees each.

These 5 non-redundant prognostic variables were: total number of embryos, the rate of cleavage arrest, the number of 8-cell embryos, day 3 FSH level, and the number of 8-cell embryos transferred. In each model, removal of each of the first four variables while keeping all other parameters constant, increased the error rate of the model, thus confirming their significant contribution. However, removal of the number of 8-cell embryos transferred, did not alter the error rate (data not shown), which suggested that this variable was less relevant than others.

24 models representing all possible combinations of these 5 variables were analyzed by CART to further define thresholds that have prognostic significance. The top 5 trees generated by CART had superior prediction scores (0.6828 to 0.6950) compared to the rest of the models (0.565 to 0.6700). As there were shared features, these top 5 tree models utilize mostly the same threshold conditions for 4 variables, while the variable, number of 8-cell embryos transferred, was not utilized by any of these top models. Therefore, IVF cycle outcomes could be most accurately predicted at ~70% by using only four non-redundant variables that are more informative than age, clinical diagnoses, or any measures of the transferred embryos.

In order to understand which factors amongst patient characteristics, diagnoses, and IVF treatment characteristics, in turn determined these four non-redundant, cohort-specific prognostic variables, tree models were constructed by MART to represent the dependence of each of these four prognostic variables. The total number of embryos, day 3 FSH, and the number of 8-cell embryos depended on and thus captured the effects of many other variables (see SI Figure 1). In contrast, the rate of cleavage arrest was independent of any of those known variables.

**SI Figure Legends and Tables.**

SI Figure 1. Variables and their relative importance in determining A) number of 8-cell

embryos, B) day 3 FSH, and C) the total number of embryos.

SI Table 1. Correlation between each pair of variables. (See separate file.)

SI Table 2. Models generated by MART® to identify non-redundant, prognostic variables.

| **Model** | **Degree of Interaction** | **Learning Rate*** | **Tree Number**^†^ | **Cross-validated (CV) Prediction Error Rate**^††^ |
| --- | --- | --- | --- | --- |
| 1 | 2 | 0·0100 | 2 | 0·300800 |
| 2 | 3 | 0·0010 | 3 | 0·300800 |
| 8 | 20 | 0·0010 | 4 | 0·308300 |
| 6 | 10 | 0·0001 | 4 | 0·315800 |
| 3 | 4 | 0·1000 | 30 | 0·323300 |
| 4 | 5 | 0·0020 | 16 | 0·323300 |
| 5 | 6 | 0·0100 | 16 | 0·330800 |
| 7 | 15 | 0·0001 | 3 | 0·330800 |

***** One of the “boosting” tuning parameters in MART® to prevent “over-fitting” of the data^2^

^†^ Number of trees constructed by MART®

^††^ 10-fold cross validation error

**REFERENCES**

1. Veeck L (1999) An Atlas of Human Gametes and Conceptuses. New York: Parthenon Publishing. 47-50 p.

2. Fonarow GC, Adams KF, Jr., Abraham WT, Yancy CW, Boscardin WJ (2005) Risk stratification for in-hospital mortality in acutely decompensated heart failure: classification and regression tree analysis. Jama 293: 572-580.

3. Friedman J (1999) Greedy function approximation: A stochastic boosting machine. Technical Report, Department of Statistics, Stanford University.

4. Friedman J (1999) Stochastic gradient boosting. Technical Report, Department of Statistics, Stanford University.

5. Friedman J (2002) Tutorial: Getting started with MART in R. Department of Statistics, Stanford University.

6. Friedman JH, Meulman JJ (2003) Multiple additive regression trees with application in epidemiology. Stat Med 22: 1365-1381.

7. Guzick DS, Overstreet JW, Factor-Litvak P, Brazil CK, Nakajima ST, et al. (2001) Sperm morphology, motility, and concentration in fertile and infertile men. N Engl J Med 345: 1388-1393.

8. Pilote L, Miller DP, Califf RM, Rao JS, Weaver WD, et al. (1996) Determinants of the use of coronary angiography and revascularization after thrombolysis for acute myocardial infarction. N Engl J Med 335: 1198-1205.
