# Supplementary material for: Defining Human Embryo Phenotypes by Cohort-Specific Prognostic Factors
Source: PLoS One. 2008 Jul 2;3(7):e2562. doi: 10.1371/journal.pone.0002562 (PMC2432022; doi:10.1371/journal.pone.0002562)
Supplement: Table S1 — Correlation between each pair of variables. (0.08 MB DOC) [file pone.0002562.s002.doc]

**SI Table 1. Correlation between each pair of variables.**

|  | Age | Gravidity | Maximum Day 3 FSH level | No. of oocytes | Average grade | Average grade of embryos transferred | No. of embryos | Percentage of 8-cell stage embryos | Percentage of embryos with ≤4 cells | Average cell no. of embryos | No. of embryos transferred | Percentage of transferred embryos at the 8-cell stage | Percentage of transferred embryos at the ≤4-cell stage | Average cell no. of embryos transferred | Fertilization rate | No. of 8-cell embryos | No. of 8-cell embryos transferred |
| --- | --- | --- | --- | --- | --- | --- | --- | --- | --- | --- | --- | --- | --- | --- | --- | --- | --- |
| Age | 1.00 | 0.19 | 0.13 | -0.30 | -0.01 | 0.12 | -0.25 | 0.03 | 0.03 | 0.03 | 0.25 | -0.20 | 0.15 | -0.13 | 0.03 | -0.17 | -0.02 |
| Gravidity | 0.19 | 1.00 | 0.01 | -0.06 | -0.09 | -0.03 | -0.03 | 0.08 | -0.03 | 0.11 | 0.06 | 0.03 | 0.03 | 0.02 | 0.03 | 0.06 | 0.09 |
| Maximum Day 3 FSH level | 0.13 | 0.01 | 1.00 | -0.24 | 0.02 | 0.05 | -0.20 | -0.02 | -0.03 | 0.02 | -0.03 | -0.12 | 0.00 | -0.05 | 0.04 | -0.20 | -0.10 |
| No. of oocytes | -0.30 | -0.06 | -0.24 | 1.00 | -0.02 | -0.25 | 0.86 | 0.06 | -0.05 | 0.06 | 0.13 | 0.35 | -0.21 | 0.27 | -0.06 | 0.68 | 0.38 |
| Average grade | -0.01 | -0.08 | 0.02 | -0.02 | 1.00 | 0.84 | 0.02 | -0.35 | 0.29 | -0.39 | 0.04 | -0.24 | 0.20 | -0.28 | 0.04 | -0.20 | -0.17 |
| Average grade of embryos transferred | 0.12 | -0.03 | 0.05 | -0.25 | 0.84 | 1.00 | -0.28 | -0.34 | 0.28 | -0.36 | 0.09 | -0.42 | 0.31 | -0.41 | -0.10 | -0.39 | -0.29 |
| No. of embryos | -0.25 | -0.03 | -0.20 | 0.86 | 0.02 | -0.28 | 1.00 | 0.05 | -0.08 | 0.05 | 0.14 | 0.41 | -0.28 | 0.35 | 0.35 | 0.77 | 0.45 |
| Percentage of 8-cell stage embryos | 0.03 | 0.08 | -0.02 | 0.06 | -0.35 | -0.34 | 0.05 | 1.00 | -0.51 | 0.61 | -0.15 | 0.77 | -0.41 | 0.50 | -0.02 | 0.51 | 0.55 |
| Percentage of embryos with ≤4 cells | 0.03 | -0.03 | -0.03 | -0.05 | 0.29 | 0.28 | -0.08 | -0.51 | 1.00 | -0.83 | 0.04 | -0.41 | 0.87 | -0.70 | -0.12 | -0.32 | -0.35 |
| Average cell no. of embryos | 0.03 | 0.11 | 0.02 | 0.06 | -0.39 | -0.36 | 0.05 | 0.61 | -0.83 | 1.00 | -0.03 | 0.45 | -0.67 | 0.81 | 0.04 | 0.35 | 0.39 |
| No. of embryos transferred | 0.25 | 0.06 | -0.03 | 0.13 | 0.04 | 0.09 | 0.14 | -0.15 | 0.04 | -0.03 | 1.00 | -0.22 | 0.02 | -0.06 | 0.16 | -0.03 | 0.39 |
| Percentage of transferred  embryos at the 8-cell stage | -0.20 | 0.03 | -0.20 | 0.35 | -0.24 | -0.42 | 0.41 | 0.77 | -0.41 | 0.45 | -0.22 | 1.00 | -0.48 | 0.58 | 0.14 | 0.64 | 0.70 |
| Percentage of transferred embryos at the ≤4-cell stage | 0.15 | 0.03 | 0.00 | -0.21 | 0.20 | 0.31 | -0.28 | -0.41 | 0.87 | -0.67 | 0.02 | -0.48 | 1.00 | -0.80 | -0.22 | -0.34 | -0.41 |
| Average cell no. of embryos transferred | -0.13 | 0.02 | -0.05 | 0.27 | -0.28 | -0.41 | 0.35 | 0.50 | -0.70 | 0.81 | -0.06 | 0.58 | -0.80 | 1.00 | 0.20 | 0.44 | 0.48 |
| Fertilization rate | 0.03 | 0.03 | 0.04 | -0.06 | 0.04 | -0.10 | 0.35 | -0.02 | -0.12 | 0.04 | 0.16 | 0.14 | -0.22 | 0.20 | 1.00 | 0.24 | 0.19 |
| No. of 8-cell embryos | -0.17 | 0.06 | -0.20 | 0.68 | -0.20 | -0.39 | 0.77 | 0.51 | -0.32 | 0.35 | -0.03 | 0.64 | -0.34 | 0.44 | 0.24 | 1.00 | 0.59 |
| No. of 8-cell embryos transferred | -0.02 | 0.09 | -0.10 | 0.38 | -0.17 | -0.30 | 0.45 | 0.55 | -0.35 | 0.39 | 0.39 | 0.70 | -0.41 | 0.48 | 0.19 | 0.59 | 1.00 |
