# Supplementary figures and images for: Defining Human Embryo Phenotypes by Cohort-Specific Prognostic Factors
Source: PLoS One. 2008 Jul 2;3(7):e2562. doi: 10.1371/journal.pone.0002562 (PMC2432022; doi:10.1371/journal.pone.0002562)

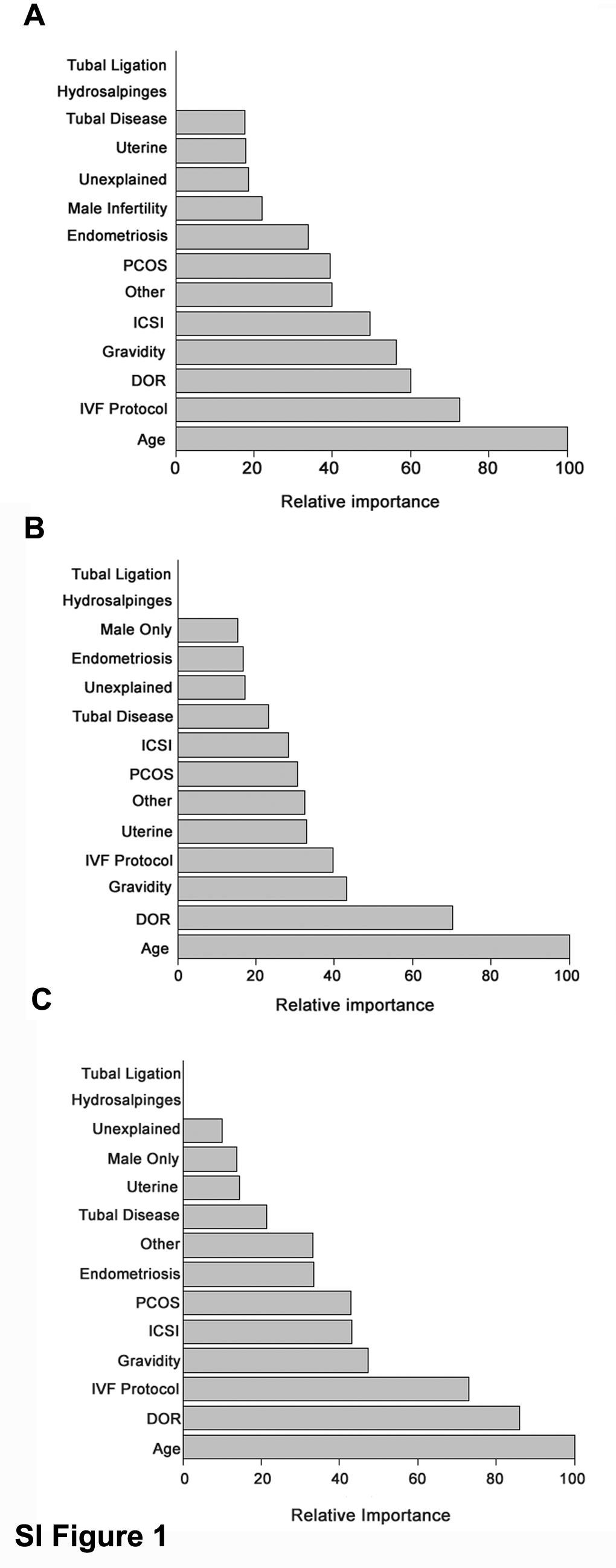

Supplement: Figure S1 — Variables and their relative importance in determining A) number of 8-cell embryos, B) day 3 FSH, and C) the total number of embryos. (2.65 MB TIF) [file pone.0002562.s003.tif]
